# Supplementary material for: Multiplexed temporally focused light shaping through a gradient index lens for precise in-depth optogenetic photostimulation
Source: Sci Rep. 2019 May 20;9:7603. doi: 10.1038/s41598-019-43933-w (PMC6527563; doi:10.1038/s41598-019-43933-w)
Supplement: Supplementary file 1 — Supplementary Information [file 41598_2019_43933_MOESM1_ESM.docx]

**Supplementary Information**

**Multiplexed temporally focused light shaping through a GRIN lens for precise in-depth optogenetic photostimulation**

Nicolò Accanto^1,2,+^, I-Wen Chen^1,2,+^, Emiliano Ronzitti^1,2, +^, Clément Molinier^1,2^, Christophe Tourain^1,2^, Eirini Papagiakoumou^1,2^ and Valentina Emiliani^1,2,*^

^1^ Wavefront-Engineering Microscopy group, Neurophotonics Laboratory, CNRS UMR8250, Paris Descartes University, 45 rue des Saints-Pères, Paris, France

^2^ Institut de la Vision, Sorbonne Université, Inserm S968, CNRS UMR7210, 17 Rue Moreau, 75012 Paris, France

^+^ Equally contributing authors

^*^ Corresponding author, valentina.emiliani@inserm.fr

**Supplementary Figure 1. Characterization of the axial FWHM of a holographic spot in the FOV of the GRIN lens**

­­­

a) Axial FWHM of one holographic spot displaced along the temporal focusing (*x*) or the perpendicular (*y*) direction (see also Fig. 2 of the main manuscript). The data are the average over 6 different repetitions of the same experiment and are given with an error bar calculated as the standard deviation over all the repetitions. The solid curves are second order polynomial fits to the data. b) Axial FWHM of one holographic spot at the coordinate (*x,y*) = (0,0) displaced along the *z* direction. The solid curve is a third order polynomial fit to the data. c) Axial FWHM of holographic spots close to the edges of the FOV in *x,y,z*. Shown are data at three different planes, namely *z*=-150 µm, *z*=0 µm and *z*=150 µm. At each plane we plot the mean value and standard deviation obtained at the positions (*x,y*)=(±60, 0), (*x,y*)=(0, ±60) and (*x,y*)=(0, 0). The data at *x*=60 µm and *x*=-60 µm, as well as those at *y*=60 µm and *y*=-60 µm are averaged together, as the FWHM is almost symmetric for positive and negative values of *x,y,z*, as the graphs a) and b) show. d,e) Analytical calculation of the axial resolution (FWHM) of holographic spots at two different planes (z=0 µm or z=± 150 µm), for different *x,y* values based on the fit results of (a,b). As the fits in (a,b) are almost symmetrical for positive and negative values of *x,y,z* we dropped the small linear terms in *x,y,z* in the fit results and retained only the quadratic terms. We considered the available FOV as the area where the FWHM is better than 35 µm, thus enabling near single cell resolution. The FOV is highly elongated, stemming from the asymmetry between the temporal focusing (TF) direction and its perpendicular direction. The circle superimposed to the plots represents the accessible diameter of the GRIN lens. Considering the GRIN lens diameter and its internal magnification of 2.6, the maximum achievable FOV has a diameter of ~190 µm, so the coordinate of a 15-µm holographic spot has to lie within a circle of diameter ~ 175 µm. As a conclusion, with MTF-CGH, in the *y* direction the FOV is limited by the GRIN lens diameter, whereas in the *x* direction, aberrations severely affect TF, limiting the FOV in *x* to a smaller area, i.e. ~ ± 50 µm from the center.

**Supplementary Figure 2. Axial confinement as a function of *x, y* and *z* for MTF-CGH spots**

Complete analysis of the 28 holographic spots shown in Fig. 2 of the main manuscript. a-c) FWHM as a function of the *x,y* and *z* axis respectively. The *x*-axis corresponds to the temporal focusing dispersion direction. In the three graphs *x,y,z* coordinates for the same spot are identified by the same colour. Comparing the three graphs one sees that the spots located at the edges of the FOV in the *x* direction (close to x=± 60 µm in a) tend to have a worse axial resolution than the others. In the *y* direction instead, it is possible to have axial resolutions ~20 µm even at the edges of the FOV, i.e. close to y=± 60 µm.

**Supplementary Figure 3. Lateral views of 28 MTF-CGH spots along *x-z* and *y-z.***

a) Same as Fig. 2c of the main manuscript, namely lateral view of 28 two-photon spots in the *x-z* plane, where *x* is the direction of temporal focusing b) Same as (a) but in the *y-z* plane.

**Supplementary Figure 4. Axial confinement as a function of *x, y* and *z* for MTF-MS**

Complete analysis of the spots generated with MTF-MS shown in Fig. 3 of the main manuscript. a-c) FWHM as a function of the *x,y* and *z* axis respectively. The *x*-axis corresponds to the temporal focusing axis. Comparing the three graphs it is apparent how the axial resolution worsens especially in the TF direction when moving away from the center. As discussed in the main manuscript however, with MTF-MS one is able to obtain an overall better axial resolution than in the case of MTF-CGH, which results in a larger available FOV for MTF-MS.

**Supplementary Figure 5. Lateral views of 20 MTF-MS spots along *x-z* and *y-z.***

a) Same as Fig. 3a of the main manuscript, namely lateral view of two-photon excitation of 20 MTF-MS spots in the *x-z* plane, where *x* is the direction of temporal focusing. b) Same as (a) but in the *y-z* plane.

**Supplementary Figure 6. Axial confinement for the MTF-CGH and in vivo setups**

Comparison of the axial profiles for a single holographic spot at (*x,y,z*)=(0, 0,0) generated with the MTF-CGH setup and with the setup for *in vivo* measurements. In the former case the axial resolution is slightly better than in the latter, which can come from several factors. One possibility is that, while in the MTF-CGH we used two SLMs to correct for aberrations at (*x,y,z*)=(0, 0,0), with the second SLM refining the corrections applied by the first one, in the setup for *in vivo* experiments we only used one SLM, therefore performing a less accurate aberration correction.

**Supplementary Figure 7. Cellular selectivity of holographic stimulation through GRIN lens in vivo.**

The 2P image (left) shows a merged average intensity projection from red and green channels at a depth of ~250 mm (i.e. with the GRIN lens in contact with the brain surface). Bright horizontal bands result from artefactual background from the photo-stimulation laser. Red circles denote target cells. In one trial (middle) where 6 target cells were simultaneously activated, cell 7 remained not activated. Cell 7 became activated in the other trial (right) when it was holographically targeted along with cells 1-6. Red vertical bars corresponds to the 3 photostimulation epochs.

**Supplementary Figure 8. Activation probability of target and non-target cells in relation to distance to target in single-cell activation experiments in vivo.**

As in Fig. 4b, the three panels represent activation probability of target and non-target cells vs. the radial distance to target (left), the x-distance to target (middle), and the y-distance to target (right). Color lines (except red) represent activation probability of target and non-target cells vs. the distance to target cell for 4 FOV of single-cell activation. Legends label the respective radial distance, x-distance and y-distance to the center FOV for target cell. Red triangles with error bars as mean±s.e.m. Red solid lines represent the exponential fits of activation probability. Two dash lines in each activation condition denote the fit-deduced distance where non-target show 50% and 10% target cells’ activation probability at threshold illumination intensity. The distance between non-target and target cell is distributed in 20-µm bins.

**Supplementary Figure 9. Simulations of the axial FWHM of a holographic spot with TF through different depths of scattering tissue for different wavelengths and objectives.**

The simulations were obtained with the same method desciribed in Ref.^1,2^, i.e. by considering a 20 µm holographic spot propagating through a turbid medium consisting of 2 µm dielectric spheres randomly distributed, having a refractive index higher by 0.1 than the surrounding medium and an average concentration of 1 sphere per 1000 µm^3^. The data for NA=0.9 and λ=800 nm (red curve) are given for comparison to Ref.^1,2^. One sees that using higher wavelength (i.e. λ=1030 nm, blue curve) enables increased robustness against scattering. For NA=0.9 and λ=800 nm the axial FWHM increases by a factor of ~ 4.5 between the no scattering case and a scattering depth of 300 µm. Increasing the wavelength to 1030 nm reduces this factor to 1.4 as a consequence of the increased scattering length. Finally, for NA=0.5, which corresponds to the GRIN lens NA, the axial FWHM only changes by a factor of 1.1 when propagating through 300 µm of scattering tissue.

**References**

1. Papagiakoumou, E. *et al.* Functional patterned multiphoton excitation deep inside scattering tissue. *Nature Photonics* **7,** 274–278 (2013).

2. Bègue, A. *et al.* Two-photon excitation in scattering media by spatiotemporally shaped beams and their application in optogenetic stimulation. *Biomed. Opt. Express* **4,** 2869–2879 (2013).
